# Relationship between Playing Musical Instruments and Subjective Well-Being: Enjoyment of Playing Instruments Scale

**DOI:** 10.3390/bs14090744

**Published:** 2024-08-26

**Authors:** Qian Zhang, Alexander Park, Kyung-Hyun Suh

**Affiliations:** 1 Department of Art Education, Xianda College of Economics and Humanities, Shanghai International Studies University, Shanghai 200089, China; 2411011@xdsisu.edu.cn; 2 Smith College of Liberal Arts, Sahmyook University, Seoul 01795, Republic of Korea; hjpark@syu.ac.kr; 3 Department of Counseling Psychology, Sahmyook University, Seoul 01795, Republic of Korea

**Keywords:** music, musical instrument, scale development, well-being, happiness

## Abstract

While the positive effects of listening to music on mental health and well-being have been extensively studied, the effects of enjoying playing musical instruments have rarely been examined. Many tools have been developed to measure music listening; however, tools to measure the enjoyment of playing instruments have not yet been developed. This study aimed to develop and validate a tool to measure such enjoyment and to examine its relationship with subjective well-being and happiness. Primary information about the aforementioned enjoyment were collected from fourteen Chinese laypersons, five music graduates, and five music teachers using open-ended questions. Item and exploratory factor analyses were conducted using data from 361 Chinese adults, and the reliability and validity of the scale and the relationships between the variables were analyzed using data from 277 Chinese adults. The results revealed that the three-factor model for the enjoyment of playing instruments demonstrated excellent model fit, and satisfactory internal consistency, test–retest reliability, and criterion-related validity were demonstrated for the learning/social bonds, achievement/pride, and cognitive refreshment/stimulation subscales. All subscales of enjoyment were positively correlated with subjective well-being and happiness. This study highlights the usefulness of the Enjoyment of Playing Instruments Scale (EPIS) as a measure for research, educational, and clinical use, providing a rationale for using instrument playing as a therapeutic approach to promote subjective well-being.

## 1. Introduction

Humans have been engaging with music since ancient times, using it for healing or therapeutic purposes even in the Middle Ages and earlier [1,2]. Unlike those from ancient civilizations, modern individuals benefit from technological advancements that allow them to access music anytime and anywhere [3]. Today, people use music for various purposes and enjoy it through diverse activities, including listening, singing, playing instruments, and composing [4]. The sustained popularity of these musical activities suggests that they provide significant benefits.

Chamorro-Premuzic and Furnham conceptualized the reasons people use music in their daily lives as regulating emotions, satisfying rational and cognitive needs, and using it as background [5]. Satisfying rational and cognitive needs may involve knowing, learning, and achieving something. Background music is utilized in various settings such as movies, dramas, weddings, and cafes. Emotional regulation may be connected to healing and therapeutic effects [6,7], music can induce physiological changes related to emotional responses [8,9]. This emotional regulation effect of music is often used for therapeutic purposes. Wang has further explored how adaptive music systems can be tailored to individuals’ emotional states to enhance therapeutic outcomes [10]. These systems use principles such as matching the tempo of the music to the listener’s mood (entrainment), gradually changing the music to guide the listener to a desired emotional state (the iso principle), and diverting attention from negative emotions to more positive or neutral feelings (diversion) [10].

Such effects have been harnessed for the treatment of affective and stress-induced disorders [11,12]. Music can facilitate rapid recovery from stress-induced anxiety and physiological responses [13]. A meta-analysis by Aalbers et al. concluded that music has short-term beneficial effects on depression symptoms [14]. These emotional and stress regulation effects have been extensively explored, particularly focusing on the impact of listening to music [15,16]. A recent study has shown that music therapy can be an effective intervention for individuals with personality disorders, aiding in emotional regulation and improving overall mental health [17]. Additionally, the therapeutic use of music has been demonstrated to alleviate symptoms of various mental health conditions, including depression and anxiety, by providing a non-invasive and creative approach to treatment [18].

However, it was found that there are psychological effects not only in receptive listening to music, but also in the expression of music [19,20]. Structured observations have highlighted that both receptive and expressive music experiences contribute significantly to psychological well-being, with expressive activities such as playing musical instruments fostering emotional expression and social interaction [9,21]. Thus, the current study investigates the various reasons people enjoy playing musical instruments and the degree to which they enjoy it, aiming to provide insights into enhancing emotional and mental health through expressive music experiences.

While emotional elevation or stabilization are common reasons for enjoying playing musical instruments, there are other significant factors. According to Chamorro-Premuzic and Furnham [5], people may play musical instruments to satisfy their cognitive needs. Chamorro-Premuzic and Furnham suggest that people may engage in playing musical instruments to fulfill cognitive needs [5]. The process of learning music scores, harmony, and sounds during musical training can lead to substantial cognitive achievements [22]. Furthermore, musical training has been shown to promote cognitive development [23], and learning to play an instrument can serve various social adaptive functions [24]. For instance, a study found that undergraduate students with musical training reported fewer conflicts in interpersonal relationships, underscoring the social benefits of musical engagement [25]. Engaging in music activities allows individuals to establish their identities in social contexts [26]. Adolescents, in particular, can enhance their self-esteem and self-efficacy through musical activities [27]. Additionally, lifelong musical activity has been associated with better global cognition, working memory, executive functions, language, and visuospatial abilities in older adults [28]. Therefore, this study aims to explore the diverse factors that contribute to the enjoyment of playing musical instruments.

We assumed that the enjoyment of playing musical instruments can positively impact subjective well-being and happiness. Previous studies indicate that music engagement enhances subjective well-being and happiness [29,30,31,32]. Furthermore, music therapy has been shown to significantly improve individuals’ subjective well-being and happiness [33,34,35]. This suggests that playing a musical instrument involves psychological factors that contribute to people’s overall happiness. Music therapy frequently incorporates playing musical instruments for therapeutic purposes [36,37,38]. Moreover, regular musical practice has been linked to improved emotional regulation, which is a key component of well-being [39]. Subjective well-being is a psychological variable that represents an individual’s happiness and can be defined as high satisfaction with one’s life, high positive emotions, and low negative emotions [40]. Structured observations and self-report measures consistently highlight the role of musical engagement in enhancing life satisfaction and reducing negative affects [41]. This study analyzes the relationship between people’s enjoyment of playing musical instruments and their subjective well-being and happiness, aiming to contribute to the broader understanding of music’s therapeutic potential.

However, although tools to measure music listening or choir activities exist [15,16,42], a valid scale to measure the enjoyment of playing instruments has not yet been developed. To examine the relationship between the enjoyment of playing musical instruments and subjective well-being and happiness, it is essential to develop a valid scale to measure this enjoyment. Therefore, this study aimed to develop a tool that properly measures the reasons why people enjoy playing musical instruments and to analyze how this enjoyment correlates with subjective well-being and happiness. This effort helps conceptualize the characteristics and behaviors associated with the enjoyment of playing musical instruments and provides useful tools for measurement. Furthermore, the research indicates that musical engagement can have significant cognitive and emotional benefits, which underscores the importance of accurately measuring enjoyment in these activities [39]. This study contributes valuable knowledge about the characteristics of playing musical instruments, which should be addressed in music therapy to promote individuals’ subjective well-being and happiness.

## 2. Methods

### 2.1. Participants

To obtain primary information about the enjoyment of playing musical instruments for constructing the items of the scale, 14 Chinese laypersons, five music graduates, and five music teachers were asked open-ended questions. Among them, 13 were women and 11 were men, and their ages ranged from 21 to 40. They were asked to “report as much as possible and describe in detail the reasons or purposes for which they enjoy playing musical instruments”. The focused group interview could not be conducted due to coronavirus disease 2019.

A total of 361 adults participated in the survey for item analysis and exploratory factor analysis (EFA). Among them, 185 (51.2%) were men and 176 (48.8%) were women, and their ages ranged from 18 to 64, with an average age of 33.94 ± 11.03.

A total of 277 adults participated in a confirmatory factor analysis (CFA), internal consistency, verification of criterion-related validity, and examination of the relationship between the enjoyment of playing instruments and subjective well-being. Among them, 135 (48.7%) were men and 142 (51.3%) were women, and their ages ranged from 18 to 57, with an average of 32.29 ± 7.62. Among them, 83 (24 men, 59 women) participated in test–retest reliability verification.

### 2.2. Measures

#### 2.2.1. Preliminary Enjoyment of Playing Instruments Items

After reviewing the results of the open-ended questionnaires, a total of 50 items on the enjoyment of playing instruments were developed. The themes of preliminary items for the enjoyment of playing musical instruments included emotional savoring, cognitive enjoyment, social enjoyment, a feeling of accomplishment, and enjoyment that could not be distinguished otherwise. After two rounds of content validity verification conducted by four professors majoring in music or psychology, 34 preliminary items were selected. In this process, among the items evaluated as being too similar, the item recommended to be the most representative was selected, and those evaluated as inappropriate were modified or removed. Those selected as preliminary items had a content validity index (CVI) value of 0.75 or higher. Among the 34 preliminary items, none had response values higher than 4.5 or lower than 1.5, which were significantly different from the average [43]. No item had a skewness and kurtosis of two or more deviations from the normal distribution, and no item showed inter-correlation coefficients greater than 0.80 [44]. With the 34 items, items with factor loadings of less than 0.35 were continuously excluded from the EFA processes. Each item was rated on a five-point Likert scale (1 = strongly disagree, 2 = somewhat disagree, 3 = neutral, neither agree nor disagree, 4 = somewhat agree, and 5 = strongly agree).

#### 2.2.2. Music USE Questionnaire

To verify the criterion-related validity of the EPIS, we used the Music USE (MUSE) questionnaire developed by Chin and Richard [45]. MUSE measures how much individuals listen to music, play musical instruments, and take music lessons as well as their music engagement style; however, we used only the items that measured their music engagement style. MUSE consists of 24 items and five factors: cognitive and emotional regulation (7 items), production (9 items), social connection (3 items), physical exercise (3 items), and dance (2 items). Items were rated on a five-point Likert scale ranging from 1 (not at all) to 5 (always). The internal consistencies (Cronbach’s α) of cognitive and emotional regulation, production, social connection, physical exercise, dance, and the total items were 0.91, 0.93, 0.89, 0.85, 0.85, and 0.95, respectively.

#### 2.2.3. Music Receptivity Scale

The Music Receptivity Scale (MRS) developed by George and Ilavarasu was used to verify the criterion-related validity of the EPIS [44]. This scale originally consists of 20 items and two factors. The first factor is the affect, which is the effect of reviving emotion or emotionally affecting and arousing interest, and the second factor is attention, which is that of concentrating and preventing negative thoughts. However, only 12 items were used because this displayed a more stable factorial structure with eight items for affect and four items for attention in scale development [46]. Items were rated on a five-point Likert scale ranging from 1 (strongly disagree) to 5 (strongly agree). Cronbach’s α for affect, attention and the total MRS with 12 items were 0.74. 0.70, and 0.66, respectively.

#### 2.2.4. Use of Music Inventory

The Use of Music Inventory (UMI) developed by Chamorro-Premuzic and Furnham was used to measure the participants’ use of music in their daily lives [5]. The UMI consists of 15 items with three subscales: the emotional use (five items), rational and cognitive use (five items), and background use (five items) of music. A total of two emotional and one musical background use items were excluded from the analysis due to a low item–total correlation. Items were rated on a five-point Likert scale ranging from 1 (strongly disagree) to 5 (strongly agree). Cronbach’s αs for the emotional use, rational and cognitive use, and background use of music and the total UMI were 0.78, 0.88, 0.84, and 0.91, respectively.

#### 2.2.5. Satisfaction with Life Scale and the Emotional Frequency Test

The Satisfaction with Life Scale (SWLS) developed by Diener et al. was used [47] to measure the participants’ life satisfaction. The SWLS consists of five items rated on a seven-point Likert scale ranging from 1 (strongly disagree) to 7 (strongly agree). Cronbach’s α of these five items was 0.89. The participants’ negative and positive emotions were measured using the Emotional Frequency Test (EFT) developed by Cho and Cha [48]. The EFT asks respondents how often they have experienced positive and negative emotions over the past month and uses a seven-point scale ranging from 1 (not at all) to 7 (always). The participants responded to four positive and four negative emotions. Cronbach’s α for negative and positive emotions were 0.90 and 0.86, respectively. The subjective well-being score was calculated by subtracting the scores for negative emotions from the sum of those for life satisfaction and positive emotions [41].

#### 2.2.6. Subjective Happiness Scale (SHS)

The participants’ subjective happiness was measured using the Subjective Happiness Scale (SHS) developed by Lyubomirsky and Lepper [49]. The scale used in this study was translated by Kim [50]. This scale consists of four items rated on a seven-point scale related to the state of happiness; however, one item asking about the state of unhappiness was reverse-scored. Cronbach’s α of the items was 0.67.

### 2.3. Procedure

Before data collection, the study protocol was approved by the institutional review board (IRB approval number: SYU 2021-12-004), and we strived to conduct data collection ethically. Written informed consent was presented to all participants for the online surveys.

The participants responded to a survey for item analysis and EFA from 11–18 June 2022. The data were collected by Shanghai Shangzi Market Consulting Co., Ltd. (Shanghai, China), an online survey company.

The participants responded to a survey for CFA, the verification of criterion-related validity, and the verification of its relationship with subjective well-being between 25 June and 9 July 2022. These data were collected using the Sojump application by Shanghai Information Technology Co. (Shanghai, China), an online survey service provider. Recruitment for this survey was conducted on internet bulletin boards and SNS. To verify the test–retest reliability, respondents were asked to leave their email address. As the survey was conducted over a specific period of time rather than at a specific point in time, the interval between test and retest was not the same, but ranged from 33 days to 58 days.

### 2.4. Statistical Analysis

This study employs a quantitative methodology. Data were analyzed using the Statistical Package for the Social Sciences (SPSS) for Windows 23.0, and the Analysis of Moment Structure (AMOS) 23.0. Pearson’s product–moment correlation analysis and EFA were conducted using SPSS, and CFA was conducted using AMOS.

The goodness of fit of the CFA was examined using the standardized root mean square residual (SRMR), root mean square error of approximation (RMSEA), Tucker–Lewis index (TLI), and comparative fit index (CFI). RMSEA and SRMR smaller than 0.08 and 0.05, respectively, suggest a satisfactory and good model fit, and TLI larger than 0.95 and CFI larger than 0.90 suggest a good model fit [51]. The composite reliability (CR) and average variance extracted (AVE) were identified for convergent validity, and a CR larger than 0.70 and an AVE larger than 0.50 indicate good convergent validity [52].

## 3. Results

### 3.1. Exploratory Factor Analysis of the EPIS

The RMSEA for the EPIS was 0.052 (χ^2^ = 980.20, df = 494) and the number of factors was two, 0.045 (χ^2^ = 793.38, df = 4624). Although the difference was less than 0.01, when the number of factors was three, the RMSEA was excellent, and the TLI was above 0.90, with the number of factors as three. Therefore, we determined the number of factors for EPIS to be three.

An EFA was conducted for the 34 primarily arranged EPIS items by fixing the number of factors to four. Items with a factor loading value of less than 0.35 and items loaded on two factors with a difference in factor loading of less than 0.10 were excluded. Subsequently, EFAs were conducted in the same manner under the same conditions. Finally, seven, six, and three items were included in Factor 1, Factor 2, and Factor 3, respectively. The Kaiser–Meyer–Olkin score was 0.917 (>0.80) for the 16 items, which indicated that this sample was suitable for the factor analysis of the enjoyment of playing an instrument.

As reported in Table 1, the EFA of the EPIS indicated that the three factors accounted for approximately 56.08% of the total variance (eigenvalues > 1.0:6.23, 1.58, and 1.17, respectively). Factor 1, in which seven items described “learning and social bonds”, accounted for 38.92% of the total variance, and the factor loadings ranged from 0.407 to 0.822. Factor 2, in which six items described “achievement and pride”, accounted for an additional 9.85% of the total variance with factor loadings ranging from 0.419 to 0.720. Moreover, Factor 3, in which the three items described “cognitive refreshment and stimulation”, accounted for an additional 9.42% of the total variance of this scale, and the factor loadings ranged from 0.435 to 0.600.

### 3.2. Confirmatory Factor Analysis of the EPIS

CFA was also conducted for the EPIS. The *χ*^2^ value of the three-factor model for EPIS was 233.90 (*df* = 101, *p* < 0.001), and the goodness-of-fit index was TLI = 0.958, CFI = 0.964, SRMR = 0.040, and RMSEA = 0.069 (CI: 0.057 to 0.081). The SRMR (<0.05) was within the range of excellent model conditions, RMSEA (<0.08) was within the range of good model conditions, and TLI and CFI (>0.95) were excellent.

**Figure 1:**
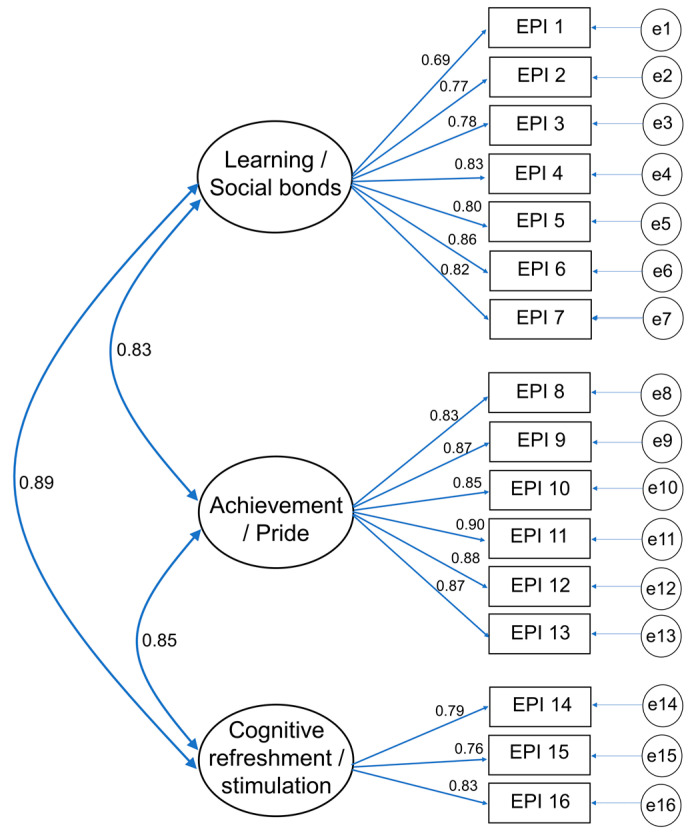
Construct model of the EPIS, three-factor model.

For the SRWs in this CFA of EPI, the learning and social bonds subscale ranged from 0.69 to 0.86 (Figure 1). Furthermore, the SRWs for the achievement and pride subscale ranged from 0.83 to 0.90. Simultaneously, the SRWs for the cognitive refreshment and stimulation subscales ranged from 0.76 to 0.83. The estimated correlation between the learning/social bonds and achievement/pride subscales, learning/social bonds and cognitive refreshment/stimulation subscales, and achievement/pride and cognitive refreshment/stimulation subscales was 0.83, 0.89, and 0.85, respectively.

CRs of the learning/social bonds, achievement/pride, and cognitive refreshment/stimulation subscales of the EPIS were 0.92, 0.95, and 0.84, respectively. The CRs of all the subscales were high (>0.70). Additionally, the AVEs of the learning/social bonds, achievement/pride, and cognitive refreshment/stimulation subscales were 0.63, 0.75, and 0.63 (>0.50), respectively.

Cronbach’s *α*s of the learning/social bonds, achievement/pride, and cognitive refreshment/stimulation subscales were 0.92, 0.95, and 0.84, respectively, while for all items for EPIS it was 0.96. The test–retest coefficients for the learning/social bonds, achievement/pride, and cognitive refreshment/stimulation subscales were 0.71, 0.60, and 0.64, respectively. The test–retest coefficient for the EPIS score was 0.72.

### 3.3. Criterion-Related Validity of the EPIS

The results of the correlational analysis of the MUSE, MRS, UMI, and EPIS are reported in Table 2. The total EPIS scores were closely correlated with the MUSE scores (*r* = 0.81, *p* < 0.001). In particular, EPIS shared 60.84% (*r* = 0.78) of the variance with the engaged production subscale of MUSE, whereas it shared only 30.25% (*r* = 0.55) with the dance subscale.

EPIS was significantly correlated with MRS (*r* = 0.50, *p* < 0.001). ELM was positively correlated with the affect subscale of MRS (*r* = 0.73, *p* < 0.001), while it was negatively correlated with the attention subscale (*r* = −0.18, *p* < 0.01). EPIS shared 53.29% of the variance with the MRS affect subscale.

EPIS was also highly correlated with UMI (*r* = 0.63, *p* < 0.001). EPIS shared 50.41% (*r* = 0.71) of the variance with the emotional use of the music subscale of UMI, and 21.16% (*r* = 0.46) with the rational and cognitive use of the music subscale.

### 3.4. Relationship between the EPIS and Subjective Well-Being

Correlational analysis revealed that EPIS scores were positively correlated with subjective well-being (*r =* 0.49, *p* < 0.001), life satisfaction (*r =* 0.59, *p* < 0.01), positive emotions (*r =* 0.57, *p* < 0.001), and negative emotions (*r =* 0.25, *p* < 0.001). According to Table 3, all EPIS subscales were positively correlated with all factors of subjective well-being and happiness. Additionally, EPIS scores were positively correlated with subjective happiness (*r =* 0.40, *p* < 0.001). All EPIS subscales were significantly correlated with subjective happiness.

## 4. Discussion

To investigate the relationship between playing musical instruments and subjective well-being, we developed a self-reported Enjoyment of Playing Instruments Scale (EPIS). The reliability and validity were verified to determine the usefulness of measuring this enjoyment. The results indicated that the EPIS is a valuable tool for quantifying enjoyment derived from playing musical instruments and exploring its positive effects. Furthermore, the enjoyment of playing instruments was found to be significantly correlated with subjective well-being and happiness. The implications of these findings are discussed below.

The 16-item EPIS displayed a stable factorial structure with the learning and social bonds, achievement and pride, and cognitive refreshment and stimulation subscales. The model fit indices for the EPIS’s factorial structure, evaluated through CFA, were excellent, affirming the three-factor model. These three factors collectively accounted for approximately 56.1% of the total variance on this scale, indicating a substantial explanatory power. The CRs and AVEs for these subscales were also excellent, underscoring the convergent validity of the three-factor EPIS model. This suggests that the items within each subscale consistently represent the underlying constructs [53]. Moreover, the EPIS exhibited strong construct validity, affirming that the scale accurately measures the intended constructs related to the enjoyment of playing musical instruments.

Given the absence of previously validated tools to measure the motivation and effects of playing musical instruments, the development of the EPIS represents a significant advancement in this field. This tool not only fills a critical gap in the existing literature, but also provides a reliable and valid measure for future research and practical applications. The EPIS can be instrumental in various contexts, such as educational settings, clinical interventions, and therapeutic practices, to assess and enhance individuals’ engagement with musical activities [39].

The learning and social bonds subscale accounted for the greatest variance in EPIS, followed by achievement and pride, and cognitive refreshment and stimulation. Notably, in this study, “learning” with items such as “I like to learn to play challenging music” and “social bonds” with items such as “I am looking forward to practicing playing musical instruments with other people” converges as a single factor. Social connection or bonding was not a factor in listening to music, but a function of playing musical instruments. Empirically, music increases individuals’ social bonds [54], which can arise while learning musical instruments [55]. The learning and social bonds subscale of the EPIS is closely correlated with the engaged production and social connection subscales of MUSE. This is evidence of the criterion-related validity of the EPIS.

Social interaction is a key component when individuals learn music together [56], and such interactions foster social connections, which are beneficial for both personal and group well-being [57]. This study suggests that people can experience a profound sense of social connection when playing instruments with others, which in turn enhances their overall enjoyment and engagement with music. The inclusion of social bonds as a significant factor in the EPIS underlines the importance of considering social contexts in musical activities.

The achievement and pride felt while playing an instrument contributed to the EPIS. Hallam also described the positive effects of an individual’s sense of achievement when learning an instrument as “the power of music” [58]. Previous studies found that playing musical instruments increases individuals’ confidence levels and positive self-concepts [59,60]. This study reinforces the idea that learning to play a musical instrument can serve as a therapeutic tool to promote self-esteem and personal growth. The achievement and pride subscale of the EPIS scale was closely correlated with the engaged production subscale of MUSE and the affect subscale of MRS. This result indicated a satisfactory criterion-related validity of the EPIS, confirming that it accurately measures the constructs associated with musical achievement and pride.

Additionally, the pride experienced in musical accomplishments can foster a positive feedback loop, where individuals are motivated to continue their musical pursuits, leading to further achievements and greater self-confidence. This cycle of positive reinforcement is particularly beneficial in educational settings, where students can experience tangible rewards for their efforts and progress [61]. This finding highlights the multifaceted benefits of musical engagement, particularly the role of achievement and pride in enhancing self-esteem and personal development.

Both expressive music activities, such as playing instruments and composing, and receptive music activities, such as listening to music and attending concerts, contribute to these positive outcomes [20,62]. Expressive activities allow individuals to actively create and interpret music, fostering a sense of accomplishment and personal expression, while receptive activities enable individuals to appreciate and emotionally connect with music [20]. These insights can inform the design of music education programs and therapeutic interventions, emphasizing the importance of fostering a sense of achievement and pride in both expressive and receptive musical activities to promote holistic well-being. By integrating both types of activities, educators and therapists can create more comprehensive and effective programs that cater to the diverse needs and preferences of individuals, ultimately enhancing their overall quality of life.

The cognitive refreshment and stimulation subscale of the EPIS closely correlated with the engaged production subscale of the MUSE. This suggests that having people play musical instruments may be utilized therapeutically to restore social relationships, promote self-esteem, improve mental health, and reduce stress responses. The is research that has found a relationship between playing musical instruments and mental health, which also supports the results of the current study [63]. The major contribution of this study is that the developed EPIS can serve as a robust tool for encouraging further research into the positive psychological effects of playing musical instruments.

Moreover, this scale provides a comprehensive framework for understanding how musical engagement can lead to cognitive benefits, such as improved memory and attention, emotional benefits, including enhanced mood and reduced anxiety, and social benefits, like stronger interpersonal connections and community engagement [64]. These findings underscore the potential of musical activities not only as a form of artistic expression, but also as a valuable component of therapeutic practices aimed at holistic well-being.

In this study, the more participants enjoyed playing musical instruments, the higher their subjective well-being and happiness. People’s enjoyment of playing instruments shared a variance of 34.8% (*r* = 0.59) with their life satisfaction. This finding suggests that playing instruments may be used as a psychotherapeutic tool for enhancing people’s life satisfaction. Thus, music therapy using musical instrument playing may effectively improve the client’s subjective well-being and happiness, particularly life satisfaction. The achievement and pride subscale in EPIS shared more variance with life satisfaction than the cognitive refreshment and stimulation subscale. This result implies that music therapy utilizing musical instrument playing could help clients feel a sense of achievement and pride, thereby enhancing self-efficacy and self-esteem. The sense of accomplishment derived from mastering an instrument provides individuals with a profound sense of purpose and fulfillment. Furthermore, the social interactions and bonds formed during group music sessions can also enhance emotional support and social well-being, contributing to overall life satisfaction [64].

The enjoyment of playing musical instruments was positively correlated with both negative and positive emotions. A mix of positive and negative emotions may exist, and some variables are positively or negatively correlated with both such emotions [65,66]. This implies that people play musical instruments when they feel both negative and positive emotions. Although a bidirectional relationship may exist between the variables, the finding that enjoying playing instruments shares more variance with positive emotions than with negative emotions highlights its potential to induce positive affect. This underscores the therapeutic value of music therapy, suggesting that engaging in musical activities can be a proactive way to boost mood and improve mental health. Music therapy, therefore, not only offers cognitive and social benefits, but also plays a crucial role in emotional regulation, making it a comprehensive approach to enhancing overall well-being.

The major contribution of this study is that the developed EPIS can serve as a robust tool for encouraging further research into the positive psychological effects of playing musical instruments. This tool can help to better understand the various dimensions of musical engagement and its impact on mental health, guiding future therapeutic practices and educational programs. By integrating the EPIS into diverse settings, practitioners can more effectively harness the benefits of musical instrument playing to promote holistic health and well-being.

## 5. Limitations of the Study

Although this study developed a useful tool to measure the enjoyment of playing musical instruments and verified the relationship between playing an instrument and subjective well-being, some limitations exist. First, the study sample is not fully representative of the global population because data collection was conducted online in China. Even so, since the people registered with the survey company and those surveyed online reside throughout China, the sample in this study is regionally representative of Chinese adults. Additionally, this study did not analyze the data by sex, age, academic majors, profession, or personal interests; further study should consider these variables as they may influence the results, which is a limitation of the current study. Second, the measures selected to verify the criterion-related validity did not accurately reflect the expressive aspects of playing musical instruments, particularly since the Music Receptive Scale focuses more on music listening. Therefore, there is a limitation in examining the criterion-related validity of the EPIS. Third, in confirmatory factor analysis, correlations between subfactors were relatively high, and further research with different samples is needed to confirm these results. Fourth, in the process of statistical analysis to meet the criteria required in psychometrics for use as a research tool, necessary items related to the enjoyment of playing musical instruments may have been excluded to maintain a stable factorial structure and satisfactory validity and reliability. Hence, developing a scale that includes various items of the enjoyment of playing musical instruments without such constraints is necessary. Finally, the causal relationship between the enjoyment of playing musical instruments and subjective well-being cannot be conclusively determined based on correlational studies rather than experimental studies.

## 6. Conclusions

This study employed a quantitative research methodology to develop and validate the EPIS, demonstrating its robustness in measuring the enjoyment of playing musical instruments. The EPIS exhibited a stable factorial structure, excellent model fit, and strong convergent and criterion-related validity, confirming its reliability and utility for researchers, educators, and music therapists. Importantly, the study revealed that the enjoyment of playing musical instruments significantly correlates with subjective well-being and happiness, thereby supporting the efficacy of music therapy that incorporates musical instruments. These findings underscore the potential of the EPIS as a valuable tool for future research and practical applications in enhancing individuals’ mental health and life satisfaction through musical engagement. However, further research with diverse samples and experimental designs is needed to explore the causal relationships and expand the scale’s applicability.

## Figures and Tables

**Table 1:** Factor structure matrix of the Enjoyment of Playing Instruments Scale (*N* = 361).

NO	Items	Factor Loadings			*h* ^2^
		1	2	3	
1.	I like learning to play challenging music.	0.553			0.401
2.	Learning to play musical instruments together with others can make me feel close to them.	0.407			0.371
3.	I like playing musical instruments as a group with other people.	0.822			0.632
4.	Playing musical instruments together improves family ties.	0.450			0.311
5.	It is fun to play musical instruments with other people.	0.822			0.620
6.	Playing musical instruments with others makes us more united.	0.516			0.473
7.	I am looking forward to practicing playing musical instruments with other people.	0.820			0.653
8.	I feel good when I realize that my musical instrument playing skills have improved.		0.599		0.445
9.	I feel good when I play an instrument in front of people and receive applause.		0.696		0.451
10.	I feel a sense of accomplishment when I play an instrument.		0.419		0.425
11.	My sense of accomplishment is great when I play an instrument well in front of others.		0.656		0.454
12.	I feel a sense of accomplishment when I master playing a piece of music.		0.601		0.420
13.	Tackling difficult music and playing it gives me a great sense of achievement.		0.720		0.518
14.	Playing an instrument makes me feel different.			0.600	0.405
15.	When I play an instrument, the thoughts that bothered me disappear.			0.435	0.383
16.	Playing musical instruments can make my partner admire me even more.			0.585	0.410
	Eigenvalues	6.23	1.58	1.17	
	% Variance	38.92	9.85	7.31	56.08

**Table 2:** Correlational matrix of MUSE, MRS, UMI, and EPIS (*N* = 277).

Scale	Learning/Social Bonds	Achievement/Pride	Cognitive Refreshment and Stimulation	EPIS
Cognitive and emotional regulation	0.69 ***	0.61 **	0.67 ***	0.71 ***
Engaged production	0.76 ***	0.68 ***	0.70 ***	0.78 ***
Social connection	0.71 ***	0.63 ***	0.63 ***	0.72 ***
Physical exercise	0.68 ***	0.65 ***	0.62 ***	0.71 ***
Dance	0.57 ***	0.44 ***	0.53 ***	0.55 ***
MUSE	0.80 ***	0.71 ***	0.74 ***	0.81 ***
Affect	0.66 ***	0.70 ***	0.64 ***	0.73 ***
Attention	−0.15 *	−0.19 **	−0.17 **	−0.18 **
MRS	0.47 ***	0.48 ***	0.44 ***	0.50 ***
Emotional use of music	0.66 ***	0.65 ***	0.65 ***	0.71 ***
Rational/Cognitive use of music	0.50 ***	0.29 ***	0.51 ***	0.46 ***
Background use of music	0.57 ***	0.45 ***	0.56 ***	0.57 ***
UMI	0.64 ***	0.49 ***	0.64 ***	0.63 ***
Skewness	−0.89	−1.35	−0.78	−1.19
Kurtosis	0.61	1.53	0.28	1.34

* *p* < 0.05, ** *p* < 0.01, *** *p* < 0.001.

**Table 3:** Correlational matrix of the EPIS and subjective well-being/happiness (*N* = 277).

Variables	Learning/Social Bonds	Achievement/Pride	Cognitive Refreshment/Stimulation	EPIS
Life satisfaction	0.57 ***	0.52 ***	0.55 ***	0.59 ***
Positive emotions	0.54 ***	0.52 ***	0.53 ***	0.57 ***
Negative emotions	0.25 ***	0.19 ***	0.29 ***	0.25 ***
Subjective well-being	0.47 ***	0.46 ***	0.43 ***	0.49 ***
Subjective happiness	0.38 ***	0.39 ***	0.34 ***	0.40 ***

*** *p* < 0.001.

## Data Availability

The datasets analyzed in this study are available from the corresponding author upon reasonable request.

## Abbreviations

EPIS	Enjoyment of Playing Instruments Scale
UMI	Use of Music Inventory
MUSE	Music Use Questionnaire
MRS	Music Receptivity Scale
SWLS	The Satisfaction with Life Scale
EFT	Emotional Frequency Test
SHS	Subjective Happiness Scale
CVI	Content Validity Index
IRB	Institutional Review Board
SPSS	Statistical Package for Social Sciences
AMOS	Analysis of Moment Structure
EFA	Exploratory Factor Analysis
CFA	Confirmatory Factor Analyses
TLI	Tucker−Lewis Index
CFI	Comparative Fit Index
RMSEA	Root Mean Square Error of Approximation
SRMR	Standardized Root Mean Square Residual
CR	Composite reliability
AVE	Average Variance Extracted
ES	Enjoyment of Singing
SRWs	Standardized Regression Weights
df	Degree of Freedom
CI	Confidence Interval
